# Validation of a prediction model for early reconnection after cryoballoon ablation

**DOI:** 10.1007/s10840-024-01811-0

**Published:** 2024-05-14

**Authors:** Kevin van Waaij, Fehmi Keçe, Marta de Riva, Reza Alizadeh Dehnavi, Adrianus P. Wijnmaalen, Sebastiaan R. D. Piers, Bart J. Mertens, Katja Zeppenfeld, Serge A. Trines

**Affiliations:** 1 Department of Cardiology, Willem Einthoven Center for Cardiac Arrhythmia Research and Management, Heart Lung Center, Leiden University Medical Center, P.O. Box 9600, 2300 RC Leiden, The Netherlands; 2 Bioinformatics Center of Expertise, Leiden University Medical Center, Leiden, The Netherlands; 3 Department of Electrophysiology, Heart Center, University of Cologne, Cologne, Germany

**Keywords:** Atrial fibrillation, Early reconnection, Dormant conduction, Cryoballoon ablation, Pulmonary vein isolation

## Abstract

**Background:**

We previously developed an early reconnection/dormant conduction (ERC) prediction model for cryoballoon ablation to avoid a 30-min waiting period with adenosine infusion. We now aimed to validate this model based on time to isolation, number of unsuccessful cryo-applications, and nadir balloon temperature.

**Methods:**

Consecutive atrial fibrillation patients who underwent their first cryoballoon ablation in 2018–2019 at the Leiden University Medical Center were included. Model performance at the previous and at a new optimal cutoff value was determined.

**Results:**

A total of 201 patients were included (85.57% paroxysmal AF, 139 male, median age 61 years (IQR 53–69)). ERC was found in 35 of 201 included patients (17.41%) and in 41 of 774 veins (5.30%). In the present study population, the previous cutoff value of − 6.7 provided a sensitivity of 37.84% (previously 70%) and a specificity of 89.07% (previously 86%). Shifting the cutoff value to − 7.2 in both study populations resulted in a sensitivity of 72.50% and 72.97% and a specificity of 78.22% and 78.63% in data from the previous and present study respectively. Negative predictive values were 96.55% and 98.11%. Applying the model on the 101 patients of the present study with all necessary data for all veins resulted in 43 out of 101 patients (43%) not requiring a 30-min waiting period with adenosine testing. Two patients (2%) with ERC would have been missed when applying the model.

**Conclusions:**

The previously established ERC prediction model performs well, recommending its use for centers routinely using adenosine testing following PVI.

**Graphical Abstract:**

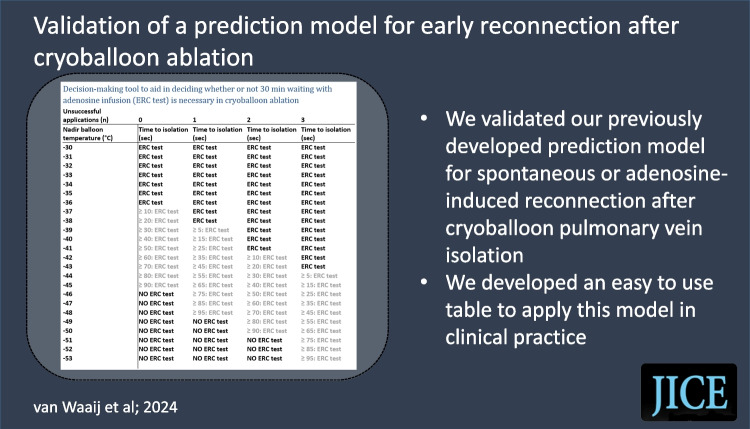

## Introduction

Cryoballoon ablation is one of the most common single-shot procedures for pulmonary vein (PV) isolation [1]. Although this technique is highly effective with a high rate of PV isolation after a single freeze [2], early reconnection during the procedure may occur. Previous research showed that the incidence of early reconnection increases in the first 30–60 min after acute PV isolation [3]. In this respect, we previously showed that incorporating a 30-min waiting period followed by infusion of adenosine to unmask dormant PV conduction (adenosine testing) increased the incidence of early reconnection after cryoballoon ablation and addressing this reconnection by additional ablation decreased atrial fibrillation (AF) recurrences [4]. Additionally, in a subsequent study, we found that the 1-year AF-free survival was similar in early reconnection/dormant conduction (ERC) patients receiving additional cryoballoon applications compared to non-ERC patients [5]. These findings signify the importance of recognizing and treating ERC.

A 30-min waiting period with subsequent adenosine administration is not without downsides. First, this adds a considerable amount of time to a routine procedure. Additionally, adenosine causes discomfort for the patient, such as chest discomfort, dyspnea, and flushing [6]. As such, predicting the absence of ERC immediately after ablation using a model based on procedural parameters instead of waiting to observe it or trying to unmask it with adenosine would be a considerable improvement.

To that end, we previously developed a prediction model for ERC which contained three independent variables [5]. From this model, a formula was constructed with a single cutoff that allowed us to predict the absence of ERC, thereby obviating the necessity for a 30-min waiting period with adenosine testing in many patients. The aim of the current study was to validate this previously developed prediction model in a new group of patients. Secondarily, we aimed to compare the 1-year AF-free survival between patients with and without ERC.

## Methods

### Study population

Consecutive patients that underwent their first cryoballoon ablation at the Leiden University Medical Center in 2018 and 2019 were retrospectively included. Baseline and procedural characteristics and follow-up data of these patients were collected using EPD-vision, the departmental cardiology information system. Biophysical parameters were obtained from cryo-applications by extracting ablation data from the ablation console using an R script (R version 4.0.2/Rstudio version 1.3.1093). All data were de-identified from personal details and anonymously stored in a Castor EDC database (https://www.castoredc.com/). Data were collected on a patient or PV level as appropriate. All patients signed an informed consent before the ablation procedure for anonymous retrospective data analysis. Due to the retrospective nature of the analysis, the ethical committee approved the study for exemption from review according to the Dutch Medical Research Involving Human Subjects Act.

### Ablation procedure

For details regarding the ablation procedure, we refer to our previous study on the prediction of ERC [5]. In short, the second- and fourth-generation cryoballoons (Medtronic Arctic Front Advance and Advance Pro, Min, USA) were used for the ablation procedure. If no isolation was achieved within 90 s, the balloon was repositioned to improve occlusion. If necessary, this procedure was repeated until isolation was achieved. Ablation time was set at our standard approach of time to isolation (TTI) + 150 s, with a maximum of 180 s in the right superior PV (RSPV), as we previously showed this to be the optimal ablation strategy [2]. The TTI was measured as the time (in seconds) between the start of an application and the moment the PV potentials disappeared or became dissociated. Following isolation, the vein was re-examined after 30 min and, if still isolated, tested with adenosine. An increasing dose of adenosine starting from 18 mg up to a maximum of 30 mg was administered intravenously until more than 1 sinus beat with blocked atrioventricular conduction was observed. ERC was defined as PV reconnection 30 min after isolation, with or without adenosine. In the case of ERC, a maximum of 2–3 additional applications were performed to abolish the ERC.

### Biophysical data collection

The freeze area under the curve (AUC) for each cryo-application was determined, where the *x*-axis was time (s) and the *y*-axis temperature (°C), for the section of the curve where the temperature was below 0 °C. The freeze magnitude for each cryo-application was determined by dividing the freeze AUC by the time (s). Finally, for the variable “warming time to 20 °C,” an actual temperature of 20 °C was often not reached before the application data ended in the console due to automatic balloon deflation. For those applications, the collected warming time was the warming time to balloon deflation.

### Follow-up

Patients were followed up on AF-free survival for at least 12 months, usually at approximately 3, 6, and 12 months after the ablation procedure. At these moments, a 24-h Holter and a 12-lead ECG were performed. Immediately after the ablation procedure, previously used antiarrhythmic drugs (AAD) were restarted. Subsequently, if no further atrial tachycardia or AF recurrence were registered, AADs were generally stopped at the first follow-up visit, unless decided otherwise by the treating physician or preferred by the patient. In addition, all patients were advised to visit the outpatient clinic when experiencing palpitations and to document the arrhythmia using 12-lead ECGs or a 24-Holter recording. Ablation success was defined as the absence of AF/atrial tachycardia on a 12-lead ECG or the absence of an episode lasting more than 30 s on a Holter/device recording, after a blanking period of 90 days. For the survival analysis, the date of the first AF/atrial tachycardia recording or the date of first typical symptoms, later confirmed by AF/atrial tachycardia recording, was selected as the recurrence date, whichever occurred first.

### Statistical analysis

All statistical tests were performed in IBM SPSS Statistics 25/29. Test results were considered statistically significant at *P* < 0.05.

#### Descriptive variables

Baseline characteristics, procedural characteristics, and biophysical parameters were compared between the ERC group and the non-ERC group using the unpaired sample *T*-test or Mann–Whitney *U* test for the continuous variables, depending on the normality of the data. Categorical variables were compared using the chi-squared test or Fisher’s exact test when applicable. Baseline characteristics were analyzed on the patient level, procedural characteristics on the patient or vein level as appropriate, and biophysical parameters on a vein level.

#### Validation of the previously developed prediction model

The previously established prediction model [5], which operates on the vein level, was evaluated by applying the previously reported formula (0.02 * TTI + 0.5 * number of unsuccessful applications + 0.2 * nadir balloon temperature) on the newly collected data. Subsequently, a receiver operating characteristics (ROC) curve was created, and the area under the ROC curve was calculated as a measure of the accuracy of the formula. Next, the sensitivity, specificity, likelihood ratios, number of veins that would be predicted to have ERC, number of ERC veins that would be missed, and predictive values of the previously established optimal cutoff value were determined. The number of veins that would be correctly predicted to have ERC was determined as follows: sensitivity * number of ERC veins. Likewise, the number of veins that would incorrectly be predicted to have ERC was determined as follows: (1-specificity) * number of non-ERC veins. The number of ERC veins that would be missed was determined by multiplying “1-sensitivity” with the number of ERC veins.

**Fig. 1:**
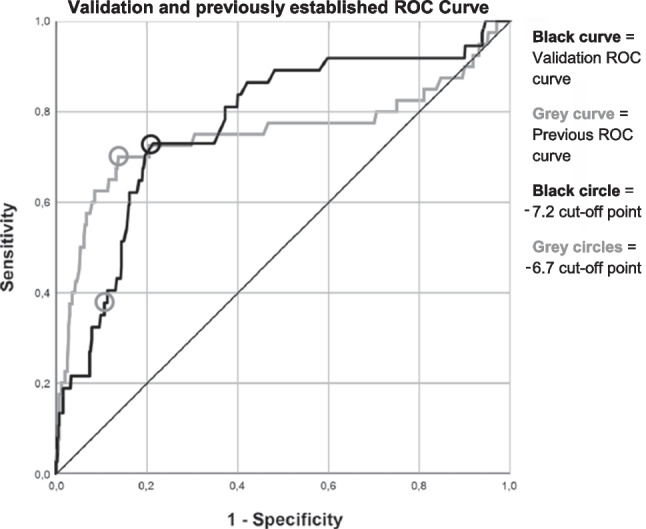
Receiver operating characteristics curves. Validation and previously constructed ROC curve. Shown here is the validation ROC curve overlayed on the previously constructed ROC curve. The validation ROC curve was also constructed using the previously developed prediction model (0.02 * TTI + 0.5 * number of unsuccessful applications + 0.2 * nadir balloon temperature). The area under the curve of the validation ROC curve is 0.773 (95% CI [0.690, 0.856]), which was 0.752 for the previous curve. An upright shift of the validation curve compared to the previous curve is visible

Next, a new cutoff value was determined to obtain similar performance in data from both the previous and present studies. This value was determined by visually comparing the coordinates of the ROC curve from the previous and present studies, searching for a cutoff that would result in a good performance in both datasets (Fig. 1). With this new cutoff, the sensitivity, specificity, likelihood ratios, number of veins that would be predicted to have ERC, number of ERC veins that would be missed, and predictive values were again determined for the data from both the previous and the present studies. Subsequently, the model with the new cutoff was applied to individual patients in the present study population, illustrating how many patients would not have required testing if the model had been available during the ablation procedures.

Moreover, an easy-to-use table was created to aid in deciding whether or not waiting and testing for ERC with adenosine is necessary during an ablation procedure. This table was created based on the new cutoff and by using different values for the parameters of the prediction model.

#### Survival analysis

Finally, 1-year Kaplan–Meier curves were constructed for the AF-free, AAD-free survival of ERC and non-ERC patients. After a blanking period of 90 days, patients entered the curve. AF-free days were not counted for the survival time if a patient was still (or again) on an AAD. A recurrence event while using an AAD resulted in censoring at the last follow-up date without AAD (events on AAD could not be counted as events without AAD but were still events, therefore leading to censoring). Patients who only had AF-free days with an AAD were not included in the Kaplan–Meier curves. The 1-year AF-free survival of ERC and non-ERC patients was compared using the log-rank test.

## Results

### Descriptives

#### Baseline characteristics

In 2018–2019, 223 patients underwent a cryoballoon AF ablation. Of these patients, 21 were excluded as cryoconsole files were unavailable and one patient because of unclear cryoconsole data, leading to a total of 201 patients for analysis. Median age was 61 years (IQR 53–69), 139 were male (69%), and 172 (86%) had paroxysmal AF. None of the collected baseline characteristics was significantly different between the ERC group and the non-ERC group (Table 1). Although no statistics were performed on the patients who were excluded from survival analysis due to continued AAD usage, the proportion of females seemed to be higher in this group.

**Table 1:** Baseline characteristics

	Overall (*n* = 201)	No ERC (*n* = 166)	ERC (*n* = 35)	*P*-value (ERC vs no ERC)	Continued AAD usage during follow-up (*n* = 30)
*Age y median, IQR*	61.0 IQR 53.0–69.0	61.5 IQR 53.0–70.0	60.0 IQR 50.0–67.0	0.199	63.5 IQR 55.0–67.5
*Male gender %, (n)*	69.2% (139)	71.1% (118)	60.0% (21)	0.197	53.3% (16/30)
*Height cm mean, SD*	179.7 ± 10.8	180.0 ± 10.9	178.2 ± 10.5	0.38	177.0 IQR 169.5–187.0
*Weight kg median, IQR*	85.0 IQR 74.0–97.5	86.0 IQR 76.0–98.0	81.0 IQR 67.0–95.0	0.079	83.0 IQR 77.3–98.3
*Body Mass Index kg/m*^*2*^*, median IQR*	26.3 IQR 23.6–29.1	26.6 IQR 23.9–29.3	25.4 IQR 22.7–28.7	0.185	26.7 IQR 23.6–29.5
*Atrial fibrillation duration y, median IQR*	3.0 IQR 1.0–6.0	3.0 IQR 1.8–6.0	3.0 IQR 1.0–6.0	0.927	3.0 IQR 1.0–5.3
*Atrial fibrillation type (paroxysmal, persistent, longstanding) %, (n)*	85.6%, 13.4%, 1.0% (172, 27, 2)	84.3%, 14.5%, 1.2% (140, 24, 2)	91.4%, 8.6%, 0% (32, 3, 0)	0.609	83.3%, 16.7%, 0% (25, 5, 0)
*Amiodaron %,(n)*	10.5% (21)	10.9% (18)	8.6% (3)	1,000	0% (0)
*Flecainide %, (n)*	33.5% (67)	32.7% (54)	37.1% (13)	0.615	13.3% (4)
*Sotalol %, (n)*	32.5% (65)	31.5% (52)	37.1% (13)	0.519	66.7% (20)
*Other AADs %, (n)*	2.0% (4)	1.2% (2)	5.7% (2)	0.142	6.7% (2)
*Previously failed AADs %, (n)*	58.7% (118)	58.4% (97)	60.0% (21)	0.864	60.0% (18)
*Electrical cardioversions before ablation %, (n)*	46.8% (94)	46.4% (77)	48.6% (17)	0.814	53.3% (16)
*Number of electrical cardioversions before index ablation n, IQR, (n)*	0 IQR 0–2 (197)	0 IQR 0–2.25 (162)	0 IQR 0–2	0.878	1 IQR 0–2
*Previous Stroke/TIA (stroke, TIA) %, (n)*	5,0%, 4,0% (10, 8)	4.8%, 3.6% (8, 6)	5.7%, 5.7% (2, 2)	0.685	6.7%, 6.7% (2, 2)
*Previous CTI ablation %, (n)*	7,0% (14)	6.0% (10)	11.4% (4)	0.273	10.0% (3)
*CHA*_*2*_*DS*_*2*_*-VASc score*	1 IQR 0–2	1 IQR 0–2	1 IQR 0–2	0.4	2 IQR 0–3
Comorbidities					
* Hypertension %, (n)*	34.8% (70)	36.7% (61)	25.7% (9)	0.213	50,0% (15)
* Dyslipidemia %, (n)*	19.5% (39)	19.4% (32)	20.0% (7)	0.934	30.0% (9)
* Diabetes %, (n)*	9.5% (19)	10.2% (17)	5.7% (2)	0.537	6.7% (2)
* Sleep apnea %, (n)*	10,9% (22)	11.5% (19)	8.6% (3)	0.772	10,0% (3)
* Coronary artery disease %, (n)*	8.5% (17)	8.4% (14)	8.8% (3)	1	10.0% (3)
* Peripheral artery disease %, (n)*	1.0% (2)	0.6% (1)	2.9% (1)	0.312	3.3% (1)
* Cerebrovascular disease %, (n)*	0.0% (0)	0.0% (0)	0.0% (0)		0.0% (0)
* Congestive heart failure %, (n)*	1.0% (2)	0.6% (1)	2.9% (1)	0.319	3.3% (1)
* Structural heart disease %, (n)*	25.9% (52)	27.7% (46)	17.1% (6)	0.194	33.3% (10)
Imaging					
* Left atrium diameter cm, median, IQR, available (n)*	4.1 IQR 3.7–4.6 (69)	4.1 IQR 3.7–4.6 (59)	4.0 IQR 3.7–4.6 (10)	0.682	4.5 IQR 3.7–4.6 (7)
* Left atrial volume index ml/m*^*2*^*, median, IQR, available (n)*	34.0 IQR 30.0–42.0 (143)	35.0 IQR 30.0–42.0 (119)	31.0 IQR 25.5–41.0 (24)	0.148	37.0 IQR 32.3–47.3 (22)
* Ejection fraction (normal, mildly abnormal,* * moderately abnormal, severely abnormal) %, (n)*	79.1%, 18.4%, 2.5%, 0% (129, 30, 4, 0)	78.7%, 19.1%, 2.2%, 0% (107, 26, 3, 0)	81.5%, 14.8%, 3.7%, 0% (22, 4, 1, 0)	0.636	76.0%, 16. 7%, 4.0%, 0% (19, 5, 1, 0)

On the patient level, baseline characteristics were compared between the ERC and non-ERC groups using Mann–Whitney *U* tests, chi-squared tests, Fisher’s exact tests, and an unpaired sample *T*-test. *IQR*, interquartile range; *ERC*, early reconnection/dormant conduction; *AAD*, anti-arrhythmic drug; *TIA*, transient ischemic attack; *CTI*, cavotricuspid isthmus.

#### Procedural characteristics

From the 201 patients, a total of 774 veins were analyzed. Four veins were excluded because of unclear data, and four veins were excluded because they did not achieve isolation using the cryoballoon. ERC occurred in 35 of the 201 patients (17.41%) and in 41 of the 774 veins (5.30%) (Table 2). Although one successful application (in case of no ERC) per PV was the aim, bonus applications were often performed at the discretion of the operator. The rate of bonus applications did not differ between the groups.

**Table 2:** Procedural characteristics

	Overall (*n* = 201 and 774)	No ERC (*n* = 166 and 733)	ERC (*n* = 35 and 41)	*P*-value
**Patient level (*n* = 201)**				
*Procedure time (min) median IQR*	165 IQR 146–190	160 IQR 142–185	180 IQR 168–209	** < 0.001**
*Total ablation time (min) median, IQR*	15.5 IQR 13.6–20.2	15.0 IQR 13.5–18.2	22.1 IQR 17.8–25.4	** < 0.001**
*Total number of applications*	5 IQR 4–7	5 IQR 4–6	7 IQR 5–8	** < 0.001**
*Balloon size (23 mm, 28 mm,* *23 & 28 mm) %, (n)*	1.0%, 99.0%, 0.0% (2, 199, 0)	0.6%, 99.4%, 0% (1, 165, 0)	2.9%, 97.1%, 0% (1, 34, 0)	0.319
*Entrance dose (mGy) median, IQR*	102 IQR 61–172	104 IQR 61–174	91 IQR 59–154	0.561
*Effective radiation dose (mSv) median, IQR*	1.6 IQR 1.0–2.8	1.6 IQR 1.0–2.8	1.8 IQR 0.9–3.1	0.978
*Fluorotime (min) median, IQR*	18 IQR 12–24 (200)	17 IQR 12–24 (165)	20 IQR 14–23	0.192
*CTI ablation %, (n)*	23.4% (47)	25.3% (42)	14.3% (5)	0.162
*Electrical cardioversion %, (n)*	28.9% (58)	32.5% (54)	11.4% (4)	**0.012**
Procedural complications				
*Stroke %, (n)*	0.0% (0)	0.0% (0)	0% (0)	
*TIA %, (n)*	1.0% (2)	0.6% (1)	2.9% (1)	0.319
*Cardiac tamponade %, (n)*	0.0% (0)	0.0% (0)	0% (0)	
*Pulmonary embolism %, (n)*	0.0% (0)	0.0% (0)	0% (0)	
*Groin complication %)*	0.5% (1)	0.60% (1)	0% (0)	1
*Phrenic nerve palsy at the end of the procedure (%)*	3.5% (7)	2.4% (4)	8.6% (3)	0.103
*Other complications (%)*	2.0% (4)	1.2% (2)	5.7% (2)	0.141
**Vein level (*n* = 774)**				
*Time to isolation (sec) median, IQR*	40 IQR 27–60	40 IQR 27–60	50 IQR 38–75)	**0.003**
*Application time (sec) median, IQR*	190 IQR 180–220	190 IQR 180–220	200 IQR 180–240	**0.024**
*Minimal esophagus temperature (°C) median, IQR*	35.7 IQR 34.3–36.1	35.7 IQR 34.3–36.1	35.5 IQR 34.6–36.3	0.764
*Unsuccessful applications %, (n)*	22.0% (170)	21.2% (155)	36.6% (15)	**0.02**
*Number of unsuccessful applications median, IQR*	0 IQR 0–0	0 IQR 0–0	0 IQR 0–1	**0.023**
*Aborted applications %, (n)*	11.0% (85)	10.6% (78)	17.1% (7)	0.199
*Number of aborted applications median, IQR*	0 IQR 0–0	0 IQR 0–0	0 IQR 0–0	0.205
*Bonus applications (done after isolation but before testing for ERC) %, (n)*	4.9%(38)	5.2% (38)	0.0% (0)	0.255
*Number of bonus applications median, IQR*	0 IQR 0–0	0 IQR 0–0	0 IQR 0–0	0.135
*Number of applications median, IQR*	1 IQR 1–2	1 IQR 1–2	2 IQR 2–4	** < 0.001**
*LSPV*	1 IQR 1–1	1 IQR 1–1	2 IQR 2–4	** < 0.001**
*LIPV*	1 IQR 1–2	1 IQR 1–1	3 IQR 2–4	** < 0.001**
*RIPV*	1 IQR 1–2	1 IQR 1–2	3 IQR 2–4	** < 0.001**
*RSPV*	1 IQR 1–1	1 IQR 1–1	2 IQR 1–2	**0.001**
*TTI more than 90 s %, (n)*	5.7% (37)	5.1% (31)	16.2% (6)	**0.014**
*LPSV (overall n* = *188, non-ERC n* = *179, ERC n* = *9)*	5.7% (9)	4.0% (6)	37.5% (3)	**0.006**
*LIPV (overall n* = *189, non-ERC n* = *176, ERC n* = *13)*	3.8% (6)	3.5% (5)	8.3% (1)	0.386
*RIPV (overall n* = *198, non-ERC n* = *187, ERC n* = *11)*	10.2% (17)	9.6% (15)	22.2% (2)	0.231
*RSPV (overall n* = *199, non-ERC n* = *191, ERC n* = *8)*	2.9% (5)	3.1% (5)	0.0% (0)	1

On the patient/vein level, procedural characteristics were compared between the ERC and non-ERC groups using Mann–Whitney *U* tests, chi-squared tests, and Fisher’s exact tests. *IQR*, interquartile range; *ERC*, early reconnection/dormant conduction; *CTI*, cavotricuspid isthmus; *TIA*, transient ischemic attack; *TTI*, time to isolation; *LSPV*, left superior pulmonary vein; *LIPV*, left inferior pulmonary vein; *RIPV*, right inferior pulmonary vein; *RSPV*, right superior pulmonary vein.

On a patient level, the procedure time was significantly longer in the ERC group than in the non-ERC group (180 IQR 168209 min vs. 160 IQR 142–185 min, *P* < 0.001). This was also the case for total ablation time (22 IQR 18–25 min vs. 15 IQR 13–18 min, *P* < 0.001), as well as for the total number of applications (7 IQR 5–8 vs. 5 IQR 4–6, *P* < 0.001). In contrast, significantly more patients from the non-ERC group had an electrical cardioversion during the ablation procedure than patients from the ERC group (33%, 54/166 vs. 11%, 4/35; *P* = 0.012).

On a vein level, TTI was significantly longer in the ERC group (50 IQR 38–75 s vs. 40 IQR 27–60 s, *P* = 0.003). This was also the case for the application time (200 IQR 180–240 s vs. 190 IQR 180–220 s, *P* = 0.024), as well as for the number of unsuccessful applications per vein (0 IQR 0–1 vs. 0 IQR 0–0, *P* = 0.023). The same was the case for the total number of applications per vein (2 IQR 2–4 vs. 1 IQR 1–2, *P* < 0.001). Finally, significantly more veins had a TTI of more than 90 s in the ERC group (16%, 6/37 vs. 5%, 31/613; *P* = 0.014).

#### Biophysical parameters

Freeze temperature at 30 and 60 s was significantly lower in the non-ERC group (respectively − 31 IQR (− 34)–(− 28) °C vs. − 28 IQR (− 32)–(− 26) °C and − 39 IQR (− 42)–(− 36) °C vs. − 34.5 IQR (− 38.0)–(− 32.0) °C; both *P* < 0.001) (Table 3). Nadir balloon temperature was also significantly lower in the non-ERC group (− 47 IQR (− 51)–(− 43) °C vs. − 41 IQR (− 45)–(− 38) °C, *P* < 0.001). Consistently, the freeze AUC and freeze magnitude were significantly larger in the non-ERC group (*P* < 0.001). Finally, the warming times to 0 °C, 15 °C, and 20 °C were significantly longer in the non-ERC group (*P* < 0.001 for all).

**Table 3:** Biophysical parameters

	Overall (n = 774)	No ERC (n = 733)	ERC (n = 41)	*P*-value
*Temperature at 30 s freeze (°C) median, IQR*	− 31 IQR (− 34)–(− 28)	− 31 IQR (− 34)–(− 28)	− 28 IQR (− 32)–(− 26)	** < 0.001**
*Temperature at 60 s freeze (°C) median, IQR*	− 39 IQR (− 42)–(− 35)	− 39 IQR (− 42)–(− 36)	− 35 IQR (− 38)–(− 32)	** < 0.001**
*Nadir balloon temperature (°C) median, IQR*	− 46 IQR (− 51)–(− 43)	− 47 IQR (− 51)–(− 43)	− 41 IQR (− 45)–(− 38)	** < 0.001**
*Temperature at time to isolation (°C) median, IQR*	− 34 IQR (− 38)–(− 30)	− 34 IQR (− 38)–(− 30)	− 34 IQR (− 37)–(− 31)	0.628
*Freeze AUC mean, SD*	7248 ± 1579	7282 ± 1567	6641 ± 1683	**0.011**
*Freeze magnitude, median, IQR* *(freeze AUC/time under 0 °C), median, IQR*	38.0 IQR 35.0–41.0	38.2 IQR 35.2–41.2	34.0 IQR 31.5–36.9	** < 0.001**
*Warming time to 0 °C (sec) median, IQR*	7 IQR 5–10	7 IQR 5–10	5 IQR 3–6	** < 0.001**
*Warming time to 15 °C (sec), median, IQR*	28 IQR 20–37	29 IQR 21–37	19 IQR 11–28	** < 0.001**
*Warming time to 20 °C (sec), median, IQR*	34 IQR 25–43	35 IQR 26–44	21 IQR 15–33	** < 0.001**

On the vein level, biophysical parameters were compared between the ERC and non-ERC groups using Mann–Whitney *U* tests and an unpaired sample *T*-test. *IQR*, interquartile range; *ERC*, early reconnection/dormant conduction; *AUC*, area under the curve.

#### Early reconnection/dormant conduction

From the 201 patients, 18 showed reconnection after 30 min waiting but before adenosine (8.96%), and 19 had dormant conduction with adenosine in one or more veins (9%). From the 774 veins, 19 had reconnection before adenosine (2%), and 23 had dormant conduction with adenosine (3%). In one patient, a LSPV received an additional application for early reconnection, after which testing with adenosine also resulted in dormant conduction.

### Validation of the previously developed prediction model

Using the previously established prediction model [5], an ROC curve was created (Fig. 1). A total of 650 veins had data for all three variables in the model and were thus included in the ROC curve, 37 of which had ERC. The area under the ROC curve was 0.773 (95% CI [0.690, 0.856]), compared to 0.75 in the derivation cohort, signifying that the model performed equally well in the validation cohort compared to the derivation cohort. However, the previously established cutoff value of − 6.7 (meaning that an outcome equal to, or above that value indicates ERC), resulted in a sensitivity of 37.84% and a specificity of 89.07% to predict ERC. The positive likelihood ratio of the model was 3.462, and the negative likelihood ratio was 0.698. The number of veins that would be correctly predicted to have ERC was 14 of the 37 ERC veins with data for all three model variables. The number of veins that would incorrectly be predicted to have ERC was 67 out of the 613 non-ERC veins with data for all three model variables. Furthermore, the number of ERC veins (with data for all three model variables) that would be missed was 23 out of the 37 ERC veins. Finally, with an ERC prevalence of 5.30% in the PVs, the positive predictive value (PPV) was 16.22%, and the negative predictive value (NPV) was 96.24%.

Because of the low sensitivity of the − 6.7 cutoff for the validation cohort presented in the current study, a new cutoff value was determined to obtain similar performances in both the derivation [5] and validation cohorts. The maneuver to change an ROC cutoff to an optimal value for both derivation and validation cohorts has been described before [7]. At a cutoff value of − 7.2 (where equal to or above − 7.2 indicates ERC), the model provided a sensitivity of 72.50% and a specificity of 78.22% in the ROC curve from the previous study (ROC curve included 421 veins, 40 with ERC and 381 without ERC). This resulted in a positive likelihood ratio of 3.328 and a negative likelihood ratio of 0.352. The number of veins that would be correctly predicted to have ERC was 29 of the 40 ERC veins with data for all three model variables. The number of veins that would be incorrectly predicted to have ERC was 83 of the 381 non-ERC veins with data for all three model variables. Furthermore, the number of ERC veins (with data for all three model variables) that would be missed was 11 out of the 40 ERC veins. Lastly, the PPV was 25.26% and the NPV 96.55%. In data from the present study, a cutoff value of − 7.2 resulted in a sensitivity of 72.97% and a specificity of 78.63%. This resulted in a positive likelihood ratio of 3.415 and a negative likelihood ratio of 0.344. The number of veins that would be correctly predicted to have ERC was 27 of the 37 ERC veins with data for all three model variables. The number of veins that would be incorrectly predicted to have ERC was 131 of the 613 ERC veins with data for all three model variables. Furthermore, the number of ERC veins (with data for all three model variables) that would be missed was 10 out of the 37 ERC veins. Lastly, the PPV was 16.04% and the NPV was 98.11%. Therefore, at this cutoff of − 7.2, the model performed similarly in data from both the previous and present study.

Subsequently, the model was applied to individual patients of the present study population. A total of 101 patients had all the necessary data available for all four veins. Out of these 101 patients, 43 received a score below − 7.2 for all four veins, thus not requiring a 30-min waiting period and adenosine testing. Out of these 43 patients, 2 patients with ERC would have been missed.

Finally, Table 4 was created based on the new cutoff to aid in deciding whether the 30-min waiting period with adenosine testing will be necessary.

**Table 4:** Decision-making tool based on new cutoff to aid in deciding whether or not ERC testing is necessary

Unsuccessful applications (*n*)	0	1	2	3
Nadir balloon temperature (°C)	Time to isolation (s)	Time to isolation (s)	Time to isolation (s)	Time to isolation (s)
− 30	**ERC test**	**ERC test**	**ERC test**	**ERC test**
− 31	**ERC test**	**ERC test**	**ERC test**	**ERC test**
− 32	**ERC test**	**ERC test**	**ERC test**	**ERC test**
− 33	**ERC test**	**ERC test**	**ERC test**	**ERC test**
− 34	**ERC test**	**ERC test**	**ERC test**	**ERC test**
− 35	**ERC test**	**ERC test**	**ERC test**	**ERC test**
− 36	**ERC test**	**ERC test**	**ERC test**	**ERC test**
− 37	** ≥ 10: ERC test**	**ERC test**	**ERC test**	**ERC test**
− 38	** ≥ 20: ERC test**	**ERC test**	**ERC test**	**ERC test**
− 39	** ≥ 30: ERC test**	** ≥ 5: ERC test**	**ERC test**	**ERC test**
− 40	** ≥ 40: ERC test**	** ≥ 15: ERC test**	**ERC test**	**ERC test**
− 41	** ≥ 50: ERC test**	** ≥ 25: ERC test**	**ERC test**	**ERC test**
− 42	** ≥ 60: ERC test**	** ≥ 35: ERC test**	** ≥ 10: ERC test**	**ERC test**
− 43	** ≥ 70: ERC test**	** ≥ 45: ERC test**	** ≥ 20: ERC test**	**ERC test**
− 44	** ≥ 80: ERC test**	** ≥ 55: ERC test**	** ≥ 30: ERC test**	** ≥ 5: ERC test**
− 45	** ≥ 90: ERC test**	** ≥ 65: ERC test**	** ≥ 40: ERC test**	** ≥ 15: ERC test**
− 46	**NO ERC test**	** ≥ 75: ERC test**	** ≥ 50: ERC test**	** ≥ 25: ERC test**
− 47	**NO ERC test**	** ≥ 85: ERC test**	** ≥ 60: ERC test**	** ≥ 35: ERC test**
− 48	**NO ERC test**	** ≥ 95: ERC test**	** ≥ 70: ERC test**	** ≥ 45: ERC test**
− 49	**NO ERC test**	**NO ERC test**	** ≥ 80: ERC test**	** ≥ 55: ERC test**
− 50	**NO ERC test**	**NO ERC test**	** ≥ 90: ERC test**	** ≥ 65: ERC test**
− 51	**NO ERC test**	**NO ERC test**	**NO ERC test**	** ≥ 75: ERC test**
− 52	**NO ERC test**	**NO ERC test**	**NO ERC test**	** ≥ 85: ERC test**
− 53	**NO ERC test**	**NO ERC test**	**NO ERC test**	** ≥ 95: ERC test**

Using different values for the parameters in the prediction model (0.02 * TTI + 0.5 * number of unsuccessful applications + 0.2 * nadir balloon temperature) and the cutoff of − 7.2, this tool was created to aid in deciding on whether or not ERC testing will be necessary. *ERC* early reconnection/dormant conduction.

### Survival analysis

**Fig. 2:**
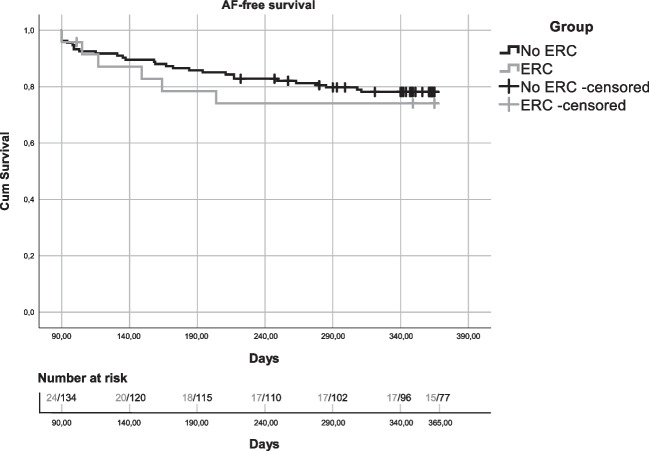
Kaplan–Meier curves of the one-year AF-free survival (without AADs) in the ERC and non-ERC group. 24 ERC patients and 134 non-ERC patients entered the curve after a 90-day blanking period. No significant difference in AF-free survival was found between the ERC and non-ERC group (*P* = 0.629). AF, atrial fibrillation; ERC, early reconnection/dormant conduction; AAD, anti-arrhythmic drug

Kaplan–Meier curves were constructed to visualize the AF-free survival without AADs in ERC and non-ERC patients (Fig. 2). From the 201 patients, 158 patients entered the curve. From the other 43 patients, 13 either had no/unclear follow-up data, 7 had no follow-up data without AAD (were using AAD after a blanking period and had no event/were censored), and 23 had a recurrence on AAD outside the blanking period before stopping the AAD. From the 158 patients that entered the curve, 35 (22.15%) had a recurrence event and 31 (19.62%) were censored before reaching 365 days. No significant difference was found in the AF-free survival between the two groups (*P* = 0.629).

## Discussion

### Main findings

The main aim of the present study was to validate the previously established ERC prediction model [5], by applying the model to data from a new group of patients. Even though the model showed a similar specificity, the sensitivity decreased substantially. However, shifting the cutoff value to a suitable value for both datasets led to a significant improvement, making the model applicable to both datasets. Secondly, no difference was found in the 1-year AF-free survival between ERC and non-ERC patients, which may signify the importance of treating early reconnection/dormant conduction with additional ablation.

### Variables in the prediction model

The previously developed prediction model contains three predictors [5]. One of these predictors is the TTI, which has more often been found to predict durable isolation [2, 8–10]. A longer TTI may be caused by incomplete occlusion of the pulmonary vein, a relation previously demonstrated by Aryana et al. [9]. An incomplete occlusion has been demonstrated to lead to more ERC and late reconnections [10–12].

The relationship between incomplete occlusion and ERC may also explain why the number of unsuccessful applications is a predictor, as we previously pointed out that unsuccessful applications may be caused by insufficient occlusion [5]. Edema occurrence after the initial application could also be an explanation, making subsequent applications less effective [13].

The final predictor is the nadir balloon temperature [5]. At different time points during the freeze, balloon temperature has often been shown to hold predictive value for ERC and late reconnection [2, 8–12, 14]. As we and others previously explained [5, 15, 16], a possible mechanism is that a better occlusion reduces warm blood leakage from the vein around the balloon, allowing lower temperatures to be reached.

### Validation of the previously developed prediction model

The area under the ROC curve (0.773) was similar to the previously found AUC of 0.752 [5]. Although the specificity found in the present study (89%) was similar, or even indicated a minor improvement over the previous specificity (86%), the sensitivity reduced substantially to 38%, compared to the 70% sensitivity from the previous study. These results indicate a similar ability of the model to identify the absence of ERC, but a decreased ability to identify the presence of ERC, implying a large reduction in the usability of the model.

With a shift in the optimal cutoff value from − 6.7 to − 7.2, the model performed equally well in both populations with reasonable sensitivity and specificity [5]. Moreover, the low ERC prevalence in PVs in data from both the previous and present study (9.22% and 5.30% respectively) resulted in very high NPVs (96.55% and 98.11% respectively). The difference in the ERC prevalence between the previous and present study could itself also be a reason for the difference in model performance. Note that the predictive values are dependent on ERC prevalence [17]. Such low occurrences of ERC in PVs have also been found in previous studies [14, 18]. The resulting high NPVs we found in our previous and present data are essential, as this indicates that a very high percentage of negative predictions will be correct, allowing 30 min to be saved in many procedures without too much risk of missing ERC. Taking the model into practice in our present study population demonstrated how well the model performs. Out of 101 patients that had all necessary data, 43 would not have required testing. Out of these 43, only 2 would have been false negative.

### Clinical implications

Confirming the absence of ERC is an important part of the current ablation procedure, as in the presence of ERC additional cryoballoon applications can reduce AF recurrences and improve AF-free survival [4, 5, 10, 19, 20]. In the present study, no difference was found between the 1-year AF-free survival of ERC and non-ERC patients, once again demonstrating the effectiveness of the additional cryoballoon applications that were performed on the ERC patients. Our prediction model allows us to predict the absence of ERC, thereby obviating a 30-min waiting period with subsequent adenosine testing in approximately 40% of patients. Therefore, applying the model during the ablation procedures would have saved 21.5 h of lab time for the whole team and avoided the discomfort of four adenosine administrations in 43 patients in this study.

Although we employ adenosine testing as the standard of care during a PVI procedure, there is conflicting evidence on the utility of this maneuver. Macle et al. [20] found that patients with dormant conduction randomized to subsequent additional ablation had significantly less recurrences with an absolute risk reduction of 27% compared to patients with dormant conduction not receiving additional applications. In contrast, Kobori et al. [21] did not find a reduction of recurrences during 1-year follow-up in patients randomly assigned to either adenosine-guided radiofrequency catheter ablation with additional ablation or radiofrequency catheter ablation without subsequent adenosine testing. Taken all data together, McLellan et al. [22] found in their meta-analysis on the role of adenosine usage after AF ablation that the induction of reconnection by adenosine reduced freedom from AF, particularly when no additional ablation was performed. They found no difference when comparing outcomes in studies of routine adenosine challenge vs no adenosine. However, they found a non-significant trend to an increase in freedom from AF in patients receiving adenosine challenge in cryoballoon ablation. Most likely, therefore, adenosine provoked reconnection decreases AF freedom, but it is less clear whether additional ablation subsequently increases this freedom. We speculate that this may be due to edema or low catheter stability in the reconnection locations. With cryoballoon, catheter stability is less of an issue than in RF ablation, and lesions are generally deeper [23], possibly overcoming edema. Therefore, we believe that adenosine testing may be beneficial in cryoballoon ablation, unless proven unnecessary by the current model.

Utilizing the previously established and now validated prediction model at a common cutoff of − 7.2 will save time and do so without missing many false negative cases. As such, its use is recommended in centers which routinely use adenosine testing following PVI.

### Limitations

The present study was a single-center study. The small size of the study populations (especially the ERC groups) of the previous and present ERC prediction study was likely partially responsible for the difference in sensitivity. Moreover, the statistics used to build the prediction model did not take into account that PVs from a single patient are not completely independent from each other. Additionally, data from the present study included 23 patients and 92 veins that were not tested for dormant conduction. Excluding these 92 veins (73 of which were used for the validation ROC curve) did not cause a relevant difference in sensitivity and specificity in the validation ROC curve at a cutoff of − 7.2 (72.22% and 79.48% instead of 72.97% and 78.63%). To make the ERC prediction model work equally well both in the derivation and the validation cohort, we had to shift the ROC cutoff to a new value for both cohorts. This is not a unique maneuver however [7], and areas-under-the-curve (AUC) were comparable between the derivation and validation cohorts, confirming the validation of the prediction model. Bonus applications may have influenced the absence or presence of ERC. The rate of bonus applications was not different between the ERC and no-ERC groups, however. Follow-up was performed with 24-h Holter recordings and 12-lead ECGs in case of symptoms. Asymptomatic episodes are therefore unlikely to have been recorded. We therefore cannot exclude that asymptomatic episodes differed between the groups. However, as 1-year rhythm outcome was not the primary outcome in this study, we believe that our primary analysis is still valid with these shortcomings. Demographic information on the patient population is not routinely collected in our departmental cardiology information system and was therefore not available for this study. Most of our patients are of Caucasian European descent. Therefore, the results of this study should be interpreted with caution for patients with other backgrounds.

### Conclusion

In the present study, the previously established ERC prediction model was validated. At a cutoff of − 7.2, the previously established ERC prediction model performed well in the study populations from both the previous and the present study. Utilizing this model will allow shorter procedure times in selected patients in centers which routinely use adenosine testing following PVI.

## Data Availability

The data that support the findings of this study are available from the corresponding author (S.T.) upon reasonable request.

## Abbreviations

PV: Pulmonary vein
AF: Atrial fibrillation
ERC: Early reconnection/dormant conduction
TTI: Time to isolation
RSPV: Right superior pulmonary vein
AUC: Area under the curve
AAD: Antiarrhythmic drugs
ROC: Receiver operating characteristics
IQR: Interquartile range
TIA: Transient ischemic attack
CTI: Cavotricuspid isthmus
LSPV: Left superior pulmonary vein
LIPV: Left inferior pulmonary vein
RIPV: Right inferior pulmonary vein
PPV: Positive predictive value
NPV: Negative predictive value

## Declarations

### Permission to reproduce material from other sources

Data was used from our previous work, which was published as open source in Heart Rhythm O2. Ownership of this material was maintained by our department. Besides this, no material from other sources was used.
